# “No one may starve in the British Empire”: Kwashiorkor, Protein and the Politics of Nutrition Between Britain and Africa

**DOI:** 10.1093/shm/hkz107

**Published:** 2019-11-04

**Authors:** John Nott

**Affiliations:** Faculty of Arts and Social Sciences (FASoS), Maastricht University, Grote Gracht 90-92, 6211 SZ Maastricht, The Netherlands

**Keywords:** malnutrition, kwashiorkor, protein, imperialism, Africa

## Abstract

Throughout the twentieth century it was widely assumed that African diets were grossly deficient in protein, that childhood protein deficiency was a natural result of this generalised diet and that a relative lack of meat and milk went some way to explaining African economic underdevelopment. This article explores why these conclusions took hold; the European deification of animal protein in previous centuries; structural changes to African diets and food economies under colonial government; and the political value of such a consensus. Unlike elsewhere in the world, where deficiency was removed from the exceptionalism of tropical medicine, protein malnutrition was constructed as a particularly African concern. Focusing this discussion on the history of the severe childhood deficiency, kwashiorkor, this article explores how the politically informed othering of African nutrition came to direct, or misdirect, the medicine of malnutrition in twentieth-century Africa.

In 1953, with the Gold Coast well on its way to independence, the British colonial government decided to release a cookbook. Intended to inform an educated readership on the relationship between good food and good health, *Gold Coast Nutrition and Cookery* epitomises the farce, tragedy and hubris which defined European attempts to influence African domesticity.^1^ Chapter 39 is, for instance, exclusively concerned with the minutiae of that mainstay of British culture, the serving and drinking of tea.^2^ While reminding the reader that ‘many doctors agree that this is an unnecessary meal’, the author goes on to describe one which is ‘dainty but light’, one in which ‘a small table and embroidered cloth is used’. Cups and saucers and teaspoons ‘should be grouped round the teapot so that the hostess may fill and hand them to each guest’. Sandwiches were seen as a suitable food for teatime, but only with the bread thinly cut and the crusts removed. In its defence, *Gold Coast Nutrition and Cookery* also covered *kenkey*, *fufu*, *tuo zaafi* and the various starchy paps and vegetable-heavy soups commonly consumed across the country. Yet, as with the more complex cultural incongruities—such as pies, tarts, soufflés and groundnut macaroons—many of the authors’ suggested sandwiches contain meat or fish or cheese. Naturally, a proper cup of tea also required milk.

Assumptions regarding the need for animal milks were also extended to infant feeding and the intimacies of African reproduction. Explaining that ‘breastmilk, even during the first 6 months of a baby’s life, does not completely supply his needs, and must be supplemented by other foods’, the authors of *Gold Coast Nutrition and Cookery* also explicitly promoted breastmilk substitutes and artificial feeding regimens for infants.^3^ Today, medical advice encourages exclusive breastfeeding for the first 6 months of life, not only because breastmilk is optimal for child development but also because bottle-feeding increases the risk of gastric infection, impeded nutrient absorption and acute malnutrition. During the early twentieth century, however, bottle-feeding boomed across the colonial world; the promotion of supplementary foods combining with increased demands on maternal time and the general devaluation of domestic *reproduction* vis-à-vis capitalist *production*.^4^ These pressures were most acute in centres of colonial commerce. In the area around Kampala, for instance, the proportion of children receiving supplementary bottle-feeds before 6 months of age increased from 14 per cent in 1950–52 to 42 per cent only 10 years later.^5^ At the same time, the amount of formula needed to adequately replace breastmilk for one child cost one-third of a labourer’s salary.^6^ The intersection of economic pressures and medical cues combined to foster ‘bottle-feeding-diarrhoea syndrome’ and new epidemics of malnutrition by the mid-century.^7^

These developments did not go unchallenged. As early as 1939, Cicely Williams had publicly derided the promotion of breastmilk substitutes as ‘murder’ and ‘the most criminal form of sedition’.^8^ In 1972, Dick Jelliffe termed this phenomenon ‘commerciogenic malnutrition’.^9^ The aggressive marketing of breastmilk by foreign multinationals—including by Nestlé, who were then accused of dressing employees as nurses and operating from maternity wards—led to increased public scrutiny, the beginnings of the Nestlé boycott in the early 1970s and the World Health Organisation (WHO)’s 1981 adoption of the International Code of Marketing of Breast-milk Substitutes. Infant malnutrition remains a pressing problem and similar critiques remain relevant, repeated and updated in view of changes to food science, food economics and medical preoccupation.^10^

Protein, and its apparent absence, lies at the heart of this enduring history. As does the prescience of Cicely Williams. During the early 1930s, and prior to her rallying against breastmilk substitution, Williams had worked as a paediatrician in the Gold Coast Medical Service. While in Accra she published two papers on a curious infantile illness, one ‘in which some amino acid or protein deficiency cannot be excluded’ as its primary cause.^11^ Taking ‘kwashiorkor’, the name used by Accra’s indigenous Ga for a set of symptoms which included oedema in the abdomen and extremities as well as changes in behaviour and in skin and hair pigmentation, Williams added an acute form of protein malnutrition to the pantheon of single-nutrient deficiencies which had emerged with the science of nutrition in the nineteenth century.^12^ From this point until past the end of empire, kwashiorkor dominated research into and discourses around food and health in Africa, its apparent prevalence confirming a continent-wide ‘protein deficit’, the widespread need for protein supplementation and contributing, ironically, to the subsequent increase in clinical deficiency.^13^

The conceptual histories of kwashiorkor, protein and protein deficiency feature heavily in the history of infant health in Africa. Yet these knotted histories have not been entirely unpicked. This article takes the view that the history of kwashiorkor is inseparable from an earlier British history of nutrition; from an enduring metropolitan image of African otherness; and from the administration of an economically diverse and often fragile sub-Saharan Empire.

Although a number of studies have explored the colonial construction of good and bad nutrition, the effects of cultural racism on scientific discourse and the use biomedicine in the extension of imperial control, siting the history of protein deficiency in an earlier European history of food and health adds necessary context to such ‘postcolonial’ analyses.^14^ At the same time, a trans-imperial focus checks the tendency of postcolonial history to address a coherent and politically consistent form of ‘colonial science’.^15^ Although recognising inconsistencies in the actions of individual scientists, colony administrations and Whitehall mandarins, this article takes the view that nutritional science drew direction as well as a degree of consistency from the passive weight of European experience.

Constructed as a timeless and endemic manifestation of a continental protein deficit, concentration on kwashiorkor was born from this European history but also found favour because it naturalised a politically expedient image of Africa. Drawing on assumptions regarding the pervasion of poverty and disease in base civilisations and primeval ecologies, ‘African exceptionalism’ was an important element of the ‘civilising mission’ and a valuable justification for the continent’s colonisation.^16^ Such ideas have endured in the ‘single story’ narratives of need which continue to dominate academic writing on African health.^17^ The invention of kwashiorkor flattened both European and African histories of food and health while also contributing to a reductive construction of African alterity.

Indeed, scientific concentration on kwashiorkor meant that—in spite of a pervasive upturn in undernutrition, food insecurity and famine which accompanied the transition to colonial capitalism—generalisations regarding continental patterns of deficiency were drawn from unrepresentative areas later described as Africa’s ‘kwashiorkor belt’.^18^ The primary locus of Anglophone nutrition research (and the primary focus of this paper) oscillated between the southern Gold Coast, where Williams’ was based into the 1930s, and southern Uganda, where the UK’s Medical Research Council (MRC) housed its tropical nutrition unit from the 1940s until the 1970s. Both areas enjoyed the remarkable security of rainforest food production, a relative absence of undernutrition and a high incidence of kwashiorkor.^19^ As ‘peasant’ economies with small settler populations, colonial land alienation was also less visible in the social aetiology of deficiency. Yet spatially specific conclusions regarding kwashiorkor were readily exported around the continent. This article takes the view that kwashiorkor, as constructed in such spaces, offered valuable distance from more politically sensitive questions of food insecurity; its political utility would influence the long-term development of nutritional science.

## The Political Extension of Nutritional Science at Home and Abroad

Loosely defined, difficult to measure and poorly understood by politicians and consumers, good and bad nutrition is remarkably subjective. According to Gyorgy Scrinis, this is a fundamental aspect of the ‘nutritionist’ discourse—or the reductive concentration on individual nutrients at the expense of social interactions between food and health—which dominated twentieth-century dietetics.^20^ Under the nutritionist paradigm, appropriate amounts of individual nutrients are quantified, the relevance of personal experience is denied and the expertise of doctors, scientists and the state becomes paramount. Especially problematic in the construction of an infant deficiency, such as kwashiorkor, is that concentration on the chemical makeup of food ignores the relevance of feeding in its overall aetiology. The opacity derived from the nutritionist paradigm has meant that the concept of malnutrition is easily appropriated and prone to vulgarisation. The recent history of ‘fad’ dieting and the wildly conflicting contemporary landscape of dietary advice highlights the instability of nutritionist discourse.^21^ Although, as Worboys and Arnold have explained, it was in the colonial world that clinical manifestations of malnutrition were ‘discovered’ following the First World War, these presentations occupied the far end of a spectrum of nutritional value which had been developed and politicised in Britain.^22^

Eating has always been political and dietetics, or the implementation of a certain dietary regimen, has always reflected the ideals of a given political economy.^23^ Failure to live up to any such ideals were naturalised in clinical manifestations of deficiency. At the end of the seventeenth century, for instance, Ireland’s ‘hung’ring for the lazy root’ would be used to explain and explain away the Great Hunger 50 years later. Potatoes were ‘food for a contented slave, not for the hardy and the brave’.^24^ Made politically relevant by the metaphor of the body politic, health and virtue were bound together and promoted through a moderate but considered diet.^25^

Unprecedented social and economic change during the nineteenth century allowed for the ready incorporation of nutrition with nascent understandings of epidemiology. The Industrial Revolution had, from the mid-eighteenth century, promoted the diversification of employment, the industrialisation and globalisation of food production and widespread movements away from the land. Patterns of consumption changed rapidly in response to huge structural changes in British food economies, while the incidence of nutritional illness changed in response.^26^ Although the economy grew rapidly throughout the nineteenth century, the sporadic decline of average heights suggests that malnutrition was pervasive amongst the emergent working class.^27^ Scurvy, beriberi, rickets and pellagra were all linked to diet during these years. The discovery of vitamins during the first decades of the twentieth century granted a satisfying chemical explanation for these correlations and imbued nutritional science with considerable momentum in the medical world.^28^

It was in this context that nutritional medicine became an overtly political concern. ‘Physical deterioration’ during the later nineteenth century was so marked that height requirements for British military recruits had to be dropped by six inches between 1845 and 1901; the failure rate for turn of the century recruits was estimated to be as high as 60 per cent.^29^ Physical disparities between British and Boer infantrymen were assumed to result from the meat-heavy diets of the South African veld and, following the British army’s inauspicious display during the South African War, investments in the well-being of the poor were increasingly understood as an indirect investment in Britain’s status as a global power.^30^ In response to fears regarding Britain’s declining ‘national efficiency’, in 1904, the government established an Inter-Departmental Committee on Physical Deterioration. The Committee’s final report recommended greater state involvement in nutrition including, amongst other things, the provision of school meals.^31^ In Foucaultian terms, nutritional science contributed to emergence of ‘biopolitics’, or the paternalistic extension of state authority over the body of the individual and the collective bodies of the wider populace.^32^ By the start of the First World War, elemental nutrients had been elevated to biopolitical objects, tools by which the government might solve problems of wartime food supply and population health.^33^ Funding followed nutrition’s newfound status and, in the interwar years, nutrition-related research won around one-sixth of all MRC grants.^34^ As the primary element of human growth, protein was imbued with the greatest biopolitical capital; in 1943, Winston Churchill announced that ‘there is no finer investment for a community than putting milk into babies’.^35^

Extending a politicised concept of nutrition beyond British borders was readily accepted in the context of empire. Investigations into nutrition in European possessions overseas found favour amongst Whitehall politicians already primed to understand the physical capital of the colonised as an extension of colonial power. Stimulated by the militarism of imperial conquest, nutrition research in the colonies initially consisted of debates over what rations were necessary for the good health of white soldiers stationed in the tropics.^36^ In the mid-1920s, however, John Boyd Orr, later the first head of the FAO, and John Gilks, then head of the Kenyan Medical Service, undertook a pioneering comparison of the largely vegan diets of the Kikuyu and the meat, blood and milk-based diets of their Maasai neighbours.^37^ Although absent from their analyses, the seizure of good agricultural land by European settlers backgrounded research which Orr and Gilks hoped might ‘hasten the improvement of the physical condition of the native and to increase his importance as an economic factor’.^38^ During a 1926 visit to the Gold Coast, William Ormsby-Gore, then Under-Secretary of State for the Colonies, explained that ‘the capacity of labour … is bound up with the question of food. There are few parts of the world where the study of dietetics is more important than in Africa’.^39^ In later years, knowledge of dietetics was integrated into civil engineering projects requiring hard, physical labour. From its inception in the 1940s, the Volta River Project employed dieticians to monitor nutritional intake in view of worker’s productivity and to recommend dietary substitutions as part of the ‘human element’ necessary for the construction of the Volta Dam.^40^ As in Britain, high-protein foods had the greatest biopolitical value. Research in the Gold Coast found that, while adults in the forest-belt were not necessarily unhealthy, they were weaker than their counterparts on the coast, where fish was a more consistent element of diet.^41^ The government’s conclusion was that ‘an increased consumption of meat is desirable, especially for those engaged in hard physical labour’.^42^ Similar conclusions were drawn from the Orr and Gilks study, where calcium deficiency was seen as the primary concern and where, as with later investigations into kwashiorkor, milk was offered as a solution which would also provide an outlet for some of Britain’s milk surplus.^43^

As an extension of state authority over a given population, biopolitics is practised differently depending upon the specific priorities of a given state. So, although the nutritionist discourse emphasised ostensibly universal, scientific understandings of nutrition, nutrition in the Empire was inextricable from the politics of empire. Central to the practice of imperial rule was the distancing of the colonised ‘other’ from the metropolitan norm.^44^ Differences in diet provided valuable distance between Europeans and their colonial subjects. As part of an imperialised form of ‘tropical medicine’, nutrition helped establish a stark contrast between the peripheral ‘white man’s grave’ and the vigour and well-being of the metropole. With the Colonial Office's endorsement, in the 1890s, the London School of Hygiene and Tropical Medicine monopolised the production of health science in Britain's tropical colonies, establishing medicine as a formal element of imperial government.^45^ The spread of nutritionist dietetics and imperial biopower created an intellectual environment that attracted anthropologists and doctors working in the colonies to the ‘otherness’ of food and nutrition in the areas to which they had been posted.^46^ Such research also helped to naturalise the relative value of the colonised. In India, the wheat and dairy diets of the Sikhs and Pathans were seen as closer to European dietaries and provided scientific credence for their eugenicist designation as ‘martial races’. David McCay, Professor of Physiology at Calcutta’s Medical College, explained in 1912 that differences in diet ‘appear to be the determining factor of the several causes that go to relegate, fix and maintain the position of a people, tribe or race in the category of men’.^47^ Orr and Gilks’ primary conclusion from Kenya was a similar validation of the view that ‘the physique of tropical native races is in no way superior, and frequently much inferior to that in civilised communities’.^48^

Although already dealing in oversimplification, the more detailed racialisation of early-twentieth-century nutrition soon declined in favour of the broader generalities that cultured kwashiorkor research.^49^ In 1933, the League of Nations’ Health Organisation implored Member States to investigate the nutritional status of their colonial subjects, arguing that ‘the fact that the greater part of the population of Africa and Asia … suffers from insufficient or faulty feeding is no longer a secret, and there is more honour to be gained in attempting to improve the situation than in concealing it’.^50^ The British response began in 1936 when Colonial Secretary, J.H. Thomas, sent a circular memo to each British possession requesting information on the nutritional status of their populations, the state of nutritional research and possible ways to improve the diets of their subjects. The resulting two-volume report, *Nutrition in the Colonial Empire*, was widely publicised and distributed, its 1939 release promoted by Lord Dufferin on the BBC and Lord Hailey in *The Times*.^51^ In *The Times*, Hailey proudly announced that the report covers ‘an area of well over two million square miles and with a population … divided into countless groups having the most different food habits and customs that it is possible to imagine’.^52^ In spite of this enormous breadth, *Nutrition in the Colonial Empire* explained that;

Diseases resulting from malnutrition … prevail almost everywhere among tribal races … excess of carbohydrate, deficient of fat and first class protein and uncertain or negligible supplies of milk and green vegetable are the outstanding features.^53^

Not only was this description grossly inaccurate, even in the context of contemporary knowledge, but it understated the worth of vegetable matter, overstated the value of animal produce and created a simple dichotomy between colonial and European diets.^54^ This othering of non-European diets was the crux of colonial dietetics, providing scientific justification for the colonisation of consumption as part of the colonial project.

## Defining Kwashiorkor: The European Roots of an African Disease

Initially understood as a severe manifestation of a simple protein deficiency, kwashiorkor would, in later years, come to sit at one extreme of a spectrum of childhood malnutrition described as Protein-Energy or Protein-Calorie Malnutrition (PEM or PCM). At the other extreme is ‘marasmus’, a total-calorie deficiency synonymous with ‘undernutrition’ or ‘wasting’. Resulting from a lack of food or the inability to digest food—as in ‘bottle-feeding-diarrhoea syndrome’—marasmus is usually seen to occur in infants. Kwashiorkor is usually diagnosed in older children, usually during or after weaning. Alongside the retarded growth common across the spectrum of PEM, symptoms of kwashiorkor include oedema, changes in skin and hair pigmentation, diarrhoea, loss of appetite, irritability, lethargy, anaemia and the fatty degeneration of the liver. A visually dramatic disease with a complicated pathology and a much poorer prognosis than marasmus, kwashiorkor offered a worthy challenge for mid-century science—its treatment remained a protracted inpatient process even into the early twenty-first century. Today, dualistic explanations of PEM are not often used. Moderate or Severe Acute Malnutrition (MAM or SAM) is instead defined according to deviation away from growth standards. The telltale oedema associated with kwashiorkor still suggests a ‘severe’ or ‘complicated’ form of malnutrition.^55^

As a fundamental element of the contemporary construction of PEM, colonial conclusions regarding kwashiorkor are alive in the contemporary consensus. ‘Constructivist’ philosophies of science and medicine explain that scientific fact does not simply exist in the natural world but is instead created by consensus and maintained by a network of social, cultural and political alliances.^56^ A disease can, likewise, be understood as a social construct, an agreement to recognise a set of symptoms as a named concern.^57^ Although wary of ontological issues bound up in retrospective diagnoses as well as in the current construction of PEM, it is still worth considering why kwashiorkor was invented in Africa, and why analogous presentations had been largely ignored in Europe.^58^ Although this surely resulted from spatial and temporal variations in food supply and domestic economics, it also relates to spatially- and temporally specific understandings of food and health. In this respect, the ‘discovery’ of kwashiorkor in the 1920s and Brock and Autret’s WHO-sponsored 1952 conclusion—that kwashiorkor is ‘the most serious and widespread nutritional disorder known to medical and nutritional science’—was derived as much from the social construction of nutrition in Europe as it was from the African disease environment.^59^

In biomedical literature, the incidence of kwashiorkor is usually taken to be determined by weaning practices, and only then influenced by the food environment. It is likely that the same can also be said for Europe. The ‘danger period during weaning’ that Hebe Welbourn associated with kwashiorkor in Uganda was certainly well known in Europe—recorded since at least pre-Christian Greece.^60^ In seventeenth-century England, stunted growth, rickets, gastroenteritis and teething were all associated with weaning. ‘Teething’ was often cited as a cause of death and the ‘weaning illness’—diarrhoeal infections as a result of sudden dietary change and increased susceptibility to infection—was common enough to be regarded as ‘normal and inevitable’.^61^ In the distinct socio-medical environment of proto-industrial London, the London Bills of Mortality began to record rickets in 1634, as well as its marked increase in subsequent years. Such diagnoses likely combined a number of bone-deforming illnesses of infancy, including wasting, scurvy and kwashiorkor. This may explain the emphasis laid by other writers on the occurrence of hepatomegaly—or the enlarged liver later seen as typical in kwashiorkor patients—in cases categorised as rickets. John Graunt’s pioneering work of epidemiology, the 1662 *Observations on the Bills of Mortality*, includes discussion of rickets and its relationship with, or confusion for, ‘livergrown’, another disease recorded in the Bills.^62^ It seems that symptomatic disorders analogous with kwashiorkor were present in the children of pre- and early-modern Europe, but that contemporary medical discourse could not explain its incidence or speak to its symptoms.

Under similar social and economic pressures to those seen in colonial Africa a century later, localised food economies and the domestic economy of childrearing changed enormously throughout during the Industrial Revolution.^63^ As a result, symptoms later associated with kwashiorkor are more visible in European medical literature. By the 1700s, accounts of oedematous malnutrition were commonly listed in paediatric textbooks and, from the nineteenth century, medical attention began to explicitly address food, feeding and deficiency in the modernising economy.^64^ Symptoms later associated with kwashiorkor—such as wasting; oedema in the legs, arms and stomach; fatty livers; skin disorders; loose stools and other intestinal problems—were regularly described in irritable and apathetic children that had been weaned too early or onto insubstantial diets.^65^ In the early twentieth century, diagnoses of such disorders emphasised the overconsumption of starch, rather than a deficiency of protein. In 1909, Czerny and Keller described Mehlnährschaden, or ‘damage by starch’.^66^ In subsequent years, reports came from Europe and the USA further detailing ‘injuries produced by starch’ and ‘diseases of infants due to prolonged feeding with excess carbohydrates’.^67^

By this time, however, low-protein diets were becoming less common, at least in Western Europe. Although not often consumed by the majority of the population, animal produce was central to European perceptions of dietary value, something at least in part related to the history of class stratification. In his classic elucidation of this point, Jack Goody references Walter Scott’s *Ivanhoe*;

‘Swine is good Saxon’ said the Jester ‘but … pork, I think, is good Norman-French; and so when the brute lives, and is in charge of a Saxon slave, she goes by her Saxon name, but becomes a Norman … when she is carried to the Castel-hall.’^68^

The same being true for sheep and mutton, cows and beef, calves and veal, meat had long been an aspirational expenditure. As average income increased, the consumption of animal produce grew in tandem. The ‘democratisation’ of meat consumption over the course of the nineteenth century has been said to have constituted a ‘food revolution’ which greatly increased the relative protein content of European diets and more firmly aligned meat and health in European medicine.^69^ By the mid-1800s, the medical consensus was that meat ‘exceed[s] all other foods in nutritional power’ and access to meat became thought of as a fundamental right.^70^ Products such as Liebig's Extract of Meat, now Oxo, and Johnston's Fluid Beef, now Bovril, emerged from the 1840s in order to provide the poor with affordable animal protein, sparking the long-standing trend of marketing manufactured food-like supplements to the poor, rather than addressing shortcomings in the supply of unreconstructed food.^71^

In the years following the First World War, protein deficiencies became increasingly interesting to a medical community recently exposed to the destitution of the poor in the ghettos of Europe and in the dustbowls of North America.^72^ Primed to ascribe particular importance to protein, research suggested that ‘hunger oedema’ or ‘war dropsy’ was the result of inadequate protein intake.^73^ The question of protein in the presentation of oedema appeared to be confirmed in the later 1920s when low-protein diets produced an analogous form of kwashiorkor in white rats.^74^ It was only in the later 1940s, in the unique clinical environments of the wartime Minnesota Starvation Study and in post-war German orphanages, that oedema was seen in undernourished patients with relatively high-protein diets.^75^ Prior to this, researchers were naturally drawn to these curious, oedematous presentations of want and, in the European cultural environment, diets deficient in protein were seen to be particularly flawed.

These developments accompanied the expansion of European involvement in Africa and served to highlight the differences between the democratised European dietetic and latterly constructed ideas of the ‘average’ African diet. Early European administrators and physicians stationed in Africa highlighted the lack of meat as a chief cause of European ill-health on the continent. In the opinion of Joseph Dupuis, a long-time British administrator working in the Gold Coast, ‘many fall victim to the climate from the adoption of a course of training improperly termed prudential; viz. a *sudden* change of diet, from *ship’s fare* to a scanty sustenance of vegetable matter’.^76^ The relative disinterest in meat as a staple in Africa was also considered particularly curious. In the early 1800s, Thomas Winterbottom, a British physician stationed in Sierra Leone, noted that;

An African, who has been feasted with every delicacy which an European table can afford, yet if rice has not constituted a part of his entertainment, will say, he has had *no meat* for so long a time, and on his return home will recur to his beloved food with redoubled ardour.^77^

Linguistics clearly offers insight into the culturally specific value of food. In French, ‘meat’, *viande*, derives from the Latin, ‘to live’. For the Tallensi of north-eastern Ghana, meat is delicious, certainly, but it is not ‘food’ in the same way as porridge. Instead, meat is *valeg*, or ‘gluttony’. In this often food-insecure savannah economy, Meyer Fortes, writing in the 1930s, found that meat was shared widely while arable produce was commonly secreted away.^78^ By the 1920s, vegetable-based diets in the Gold Coast’s Akan rainforest were seen by some British observers as actively ‘dangerous’.^79^

The advent of tropical medicine facilitated the spread of nutrition research into the fertile ground of the Global South, where oedematous malnutrition was found to be particularly prevalent. In Latin America, symptoms later defined as kwashiorkor were described in a number of articles from 1908.^80^ In literature emanating from the French Empire, doctors described the ‘Swelling [disease] of Vietnam’ as early as 1913.^81^ Similar groups of symptoms were also being described in the British Empire at least by the 1920s.^82^ Yet it was the work of Cicely Williams which cemented ‘kwashiorkor’ as an illness undocumented in Western medical literature or Western epidemiology. Despite its global incidence, her construction would spark decades of debate and research into primarily African presentations of the illness.

In earlier years, however, European doctors in Africa had been relatively dismissive of such symptoms, even though they were readily apparent. In the 1870s, a German doctor travelling in the Loango Kingdom (now part of the Democratic Republic of Congo) found children with protruding abdomens, ‘just as white children, who had consumed large quantities of carbohydrate-rich food in early youth’.^83^ Early doctor-explorers like David Livingstone and Thomas Winterbottom had received their medical training in the particular nutrition environment of the Industrial Revolution. However, as Sjoerd Rijpma explains, by the 1920s it was ‘not surprising that [Williams] called it a “new disease”: the symptoms were hardly seen in Europe then’.^84^

In the absence of effective medical communication, it was not until later in the twentieth century that the numerous descriptions of kwashiorkor began to be brought together.^85^ Williams’ work was particularly attractive because it emphasised an absence of dietary protein, rather than an excess of starch. Dermatological signs—sometimes described as ‘crazy-pavement dermatitis’—were also made more dramatic by their presentation on black skin, as well as by the white-colonial obsession with blackness.^86^ In later accounts, ‘the degree to which dyspigmentation can be taken as evidence of kwashiorkor’ would be forwarded as a potential indicator ‘in studying the frequency and importance of the syndrome’ at the population level.^87^ In general, Williams’ lengthy descriptions of kwashiorkor stood out because, through the prism of British tropical medicine, this definition fit with the established otherness of African life, something which was only exacerbated by William’s use of and, subsequently, the global adoption of Ga nomenclature.

Williams’ somewhat punitive 1936 transfer to Malaya restricted her ability to work on kwashiorkor. Research, however, intensified under the industry of Hugh Trowell and the cohort collected around Kampala’s Mengo Hospital. Trowell’s progress was slow and frustrated by the complicated pathology of kwashiorkor. In 1937, after feeding patients with cow’s milk, liver and all known vitamins and continuing to lose around 40 per cent of patients, Trowell was convinced that kwashiorkor was not a simple single nutrient deficiency.^88^ The mysterious aetiology of the disease only began to unfold in 1942, after Jack Davies, Mengo’s new pathologist, showed up degenerated pancreases in post-mortem examinations. This, Trowell and Davis would go on to explain, suggested that patients were unable to digest their food due to a shortage of pancreatic secretions; it also explained why supplements failed to relieve patients in advanced cases. By 1946 it was suggested that a lack of protein could severely harm the tissues and organs of the body because it restricted the ability to create new tissue. The functioning of the liver and the pancreas were gradually undermined, leading to a decline in enzyme production and the restriction of nutrient absorption. The subsequent failure to digest led to diarrhoea and, because of excessive fat, an enlarged liver.^89^

Trowell’s research was, however, hindered by the reservations of colonial administrators. In Uganda, R.S.F. Hennessey, a politically minded pathologist, who would later become Principle Medical Officer of the Uganda Protectorate, took little interest in kwashiorkor. Prior to his promotion, Hennessey would perform a number of autopsies in the space of an hour, mainly on vital organs extracted by students and medical assistants. Jack Davies, taking 50 sections of one cadaver, found the critical pancreatic degenerations on his first attempt.^90^ It is difficult to say whether Hennessey’s failure to do more to investigate the pathology of kwashiorkor was due to incompetence or wilful ignorance. In any case, he was certainly resistant to Trowell’s investigations. Trowell describes Hennessey as saying;

Oh, there’s nothing in Kwashiorkor. It’s just that they’re not very well fed, then they pick up malaria, hookworms, and all the rest of it. What’s the mystery? There is no new complaint here.^91^

Both Hennessey and John Hall, the Governor of Uganda, tried to privately dissuade Trowell from keeping on with his kwashiorkor work, Hall once asking ‘where will all this racket end?’^92^ Citing Roger Whitehead, a long-time director of the MRC nutrition unit in Uganda and then the Gambia, Tappan suggests that, unlike previously unknown vitamin and mineral deficiencies which provided opportunities for science to improve the lives of colonial subjects, ‘protein malnutrition pointed to the poverty of colonial populations’.^93^

It is, however, important to realise that the concerns of the administrations inside individual colonies were not necessarily the same as those in Whitehall. While in-country administrators were understandably reluctant to draw attention to a high incidence of kwashiorkor, protein deficiencies were still more palatable than a fundamental lack of food, especially if they could be presented as an endemic problem of the African environment. Emphasising that ‘the native food problem is not so much one of quantity as one of quality’, Whitehall promoted a narrative in which the presence of kwashiorkor could actively alleviate metropolitan responsibility for malnutrition in the colonies.^94^ Since subsequent research concentrated on areas with highly-visible burdens of kwashiorkor and a relative lack of undernutrition, much of the resultant literature emphasised a cultural proximity to malnutrition. Accounts such as Welbourn’s late 1950s survey of Ugandan kwashiorkor patients found that no families appeared poor, while some seemed comparatively well-to-do.^95^ Earlier, in the mid-1940s, the Ugandan administration stated that ‘the majority of children in Buganda show signs of malnutrition’. This was not necessarily a problem for the government of the day. Malnutrition, the same report would explain, ‘is not due so much to absolute poverty as to ignorance, conservatism and superstition’.^96^ These were enduring and malleable conclusions that could be shaped to fit various political spaces around the continent. In 1962, under Apartheid, the South African minister of health spoke in parliament in order to explain that there was no famine or undernutrition in the country but, because of custom, ignorance and immorality, kwashiorkor was still present.^97^

Unlike kwashiorkor, hunger presented a more difficult conceptual problem for imperial administrations. Although the extension of food relief had dampened famine mortality, it is John Iliffe’s enduring generalisation that, across Africa, ‘epidemic starvation for all but the rich gave way to endemic undernutrition for the very poor’.^98^ Despite this, discourse regarding nutrition focussed not on shifting continental patterns of hunger, or *undernutrition*, but on kwashiorkor, defined as protein *malnutrition*, and enveloped in the scientific and political opacity of nutritionist discourse. While ‘malnutrition’ acknowledges some problem with the nutrient composition in an individual diet, it fails to explain precisely what is wrong.^99^ Instead, malnutrition suggests a dichotomy between good and bad diets, as well as the capacity for improvement. As a fundamental lack of nutrients, undernutrition is a much more substantive failure, one which exists beyond the nutritionist paradigm. The ready conflation of ‘undernutrition’ and 'malnutrition' can be seen in many histories of African nutrition.^100^ Such distinctions were, by contrast, recognised by imperial administrations. The construction of kwashiorkor as the clinical manifestation of a continental protein deficit offered valuable distance from any upturn in undernutrition. The history of kwashiorkor, therefore, has just as much to do with an absence of food as it does with an absence of high-protein foods.

There was a year between the receipt of replies to the 1936 Thomas circular and the final publication of *Nutrition in the Colonial Empire*. Over the course of that year, representative preliminary reports were ‘depoliticised’ by the Colonial Office to remove any suggestion that low wages, inadequate returns from cash-crops and declines in food production were complicit in the pervasive pattern of malnutrition.^101^ In the Gold Coast, as elsewhere, reports circulated after the Colonial Office’s initial request for information suggested that, contrary to the conclusions which were published later, the colony’s greatest nutritional problem was not a deficiency of quality foods (although that was a concern) but endemic undernutrition and the recurrent threat of famine in particular areas.^102^

In response to Whitehall’s request for information on colonial nutrition, the Gold Coast government seconded F.M. Purcell to undertake a detailed investigation into nutrition across the colony’s three main agro-economic regions—the dry northern savannah, the rainforest and the coastal plain. Inaction and official indifference marred Purcell’s investigation.^103^ Audrey Richards, the pioneering anthropologist and sometime colonial officer, later explained that, ‘in spite of our circulars, the Heads of the technical services (medicine, agriculture and education), do not seem to have cooperated very closely’.^104^ After 2 years and some 200 pages, Purcell’s foremost concern was that, throughout the savannah, ‘there is a severe shortage of every kind of food during several months yearly’.^105^ On the completion of his report, ‘a senior officer’ had told him that ‘“this report will not be sent home … as it reveals neglect on the part of the local administration in the Northern Territories”… unofficially it was explained to me that “no one may starve in the British Empire.”’^106^

It was, in fact, sent to London, although nearly 2 years after its completion. On receipt of the report, S. Culwick, the Nutrition Officer assigned to the Colonial Office, noted that ‘two and a half years ago the [Gold Coast] Nutrition Committee agreed to … immediate measures to meet these local shortages … it would appear that has not been followed up’.^107^ Disappointed by the official response to his work, Purcell had already resigned his position and gone to the press. ‘Venting his grievances’ in a 1943 letter to the editor of *West Africa*, Purcell’s revelations were notable because they jarred with official representations of nutritional health in the colonies.^108^ Purcell’s findings were reprinted a number of times between 1943 and 1946. In 1946, the *West African Review* took it upon itself to remind readers that ‘we must avoid the error of supposing that shortage of vital foods, present and pending, is something affecting Germans alone’.^109^ In their response, the Colonial Office explained that ‘short term, local relief measures operate for the periodical acute hunger in the villages of the territories, but the solution of the problem is a matter for long-term development’.^110^ At the behest of Oliver Stanley, the Secretary of State for the Colonies, Governor Alan Burns was asked to explain the state of nutrition in the north. Assuring Stanley that ‘I am by no means complacent about the situation which the Purcell Report reveals’, Burns stressed that ‘the problems of nutrition can only be tackled effectively on a long term basis and in conjunction with the many other problems with which we are faced’.^111^

Starvation was a difficult problem for governments to contend with. Humanitarianism had come of age and absolute failures of subsistence were widely seen as a governmental responsibility, if not a direct failure of imperial government.^112^ In his unpublished reply to the 1936 Thomas circular, the Director of the Gold Coast’s Medical Department suggested as much, since ‘so much attention has been devoted to the cocoa and mining industries … food produced on their farms is apt to be overlooked’.^113^ Similar critiques were common throughout the 1930s.^114^ In 1939, Audrey Richards summed up the opinion of many scholars, writing that the diet and health of the colonised ‘has deteriorated in contact with white civilisation rather than the reverse’.^115^ However, as Michael Worboys has explained, through publications like *Nutrition in the Colonial Empire*, Whitehall sought to reframe malnutrition from an epidemic, structural problem resulting from colonialism ‘to an endemic one for which colonialism had little responsibility and over which it could exercise little control’.^116^

The promotion of protein malnutrition as Africa’s greatest nutritional concern complemented this more general process. Constructed as a problem of ignorance and backwardness, the high incidence of kwashiorkor in Uganda never earned much consternation from London. Despite reservations from Hennessey and Hall inside the Protectorate, the research undertaken in Uganda came to be considered a boon to the British Empire. At the end of his speech inaugurating Kampala’s new Makerere Medical School in 1951, the then Colonial Secretary James Griffith announced that ‘the medical school is known throughout the whole of Africa … for the magnificent research work of Dr Hugh Trowell’.^117^ As Tappan has explained, Makerere was taken by the MRC’s chief executive, Sir Harold Himsworth, to be a ‘model for medical research in the tropics’.^118^ Although Himsworth expected Makerere to be built on universalistic understandings of health, kwashiorkor research found political favour because it also fit with exceptionalist ideas of African primitivism, African diet and the African disease environment.

This is a distinction that deserves to be stressed. Although, as Tilley has argued, we cannot speak of a coherent ‘imperial science’, the delicate politics of famine and the emotive politics of food did shape medical discourse.^119^ Research into nutrition was characterised by political and scientific dissonance at all levels of administration, but the influence of systemic pressures—not only the contemporary politics of empire but also the weight of the European past and of past scientific consensus—was borne out in the medicine of malnutrition. In the Gold Coast, Purcell’s gravest concerns were reserved for the hungry in the northern savannah. Although oedematous infant malnutrition was apparently endemic in the rainforest—‘known everywhere as “ahonhon”’—his tone is much lighter when the focus shifts to Akim;

Then to consider fruits; the ignorance and indifference to fruits is astonishing. There are oranges, bananas, pineapples, pawpaw, mango, guava &c – yet no Akan housewife would think of making a banana fritter; and they have never even heard of a pineapple soufflé!^120^

The relative abundance of food meant that there was nothing sombre to be said about nutrition here and, as in *Gold Coast Nutrition and Cookery*, the soufflé again appears as a touchstone of civility. As a problem of ignorance and indifference, malnutrition here was to be remedied with the slow march of European civilisation and the slow spread of European science.

## Colonising Kwashiorkor: Tropical Medicine and Indigenous Knowledge

As with most pre-colonial understandings of illness in Africa, infantile deficiencies were conceptualised and prevented within social frameworks.^121^ However, science and scientism—as well as the more specific correlates of nutrition and nutritionism—fundamentally undermined any such social construction of kwashiorkor. Its subsequent medicalisation would go on to mar the prevention of malnutrition at the same time as promoting commercial salves and technical treatments for a symptom of social dislocation.^122^ Cicely Williams was remarkable in part because she recognised this, listening to her patients and responding to the cultures she encountered with a degree of curiosity and sensitivity uncommon in colonial physicians.^123^ In her second paper on kwashiorkor, she began by explaining that such symptoms were understood by the Ga as ‘the disease the deposed baby gets when the next one is born’.^124^ In her MD thesis, she recognised ‘the most uncomprehending indignation, rage and bitterness in a child of three years old who found that his place on his mother’s back was suddenly usurped by a new baby’.^125^

As part of his broader disregard for Williams’ work, Hugh Stannus, then the Empire’s leading authority on nutrition, brushed the social epidemiology aside, stating that the transliteration of kwashiorkor was irrelevant and a ‘common superstition’ since, even ‘among the Wa-yao of Central Africa the disease, whatever it may be, is called *litango lya kututa* – each successive child is said to push (*kututa*) the previous one into its grave’.^126^ Operating within the nutritionist dietetic, and in view of exceptionalist ideas of African health, scientific consensus narrowly framed kwashiorkor as a simple deficiency of protein. Clearly, however, the aetiology of kwashiorkor was understood by the colonised in a very different way. In 1954, Trowell et al. listed some 32 names for kwashiorkor taken from a handful of African and Asian countries, most of which suggest the same social aetiology of the disease—short birth spacing and short breastfeeding durations.^127^ For those prone to the disease, kwashiorkor did not necessarily relate to diet but was instead tied to household makeup, conjugal responsibility and the highly personal intricacies of childrearing.

If protein deficiency was indeed endemic during the early twentieth century, it may more accurately be seen as the result of relatively recent changes to African domestic economies. Using the writings of David Livingstone and other European physician-explorers, Sjoerd Rijpma has suggested that, at least in the early nineteenth century, social and sexual tradition encouraged low birth rates and long breastfeeding durations, actively protecting children from deficiency. In *Missionary Travels*, Livingstone noted that many illnesses common in England were absent in Africa and that ‘in the more central parts the people were remarkably kind and civil and free from disease’.^128^ On the coasts, as well as in central areas under heavy pressure from the slave trade, epidemic disease was brought in by ship, while the pull of trade goods and foreign wealth stoked slaving, conflict and social disintegration. Although the generality of Rijpma’s argument undoubtedly paves over significant variations across the continent, studies of historical anthropometry have found similar statures on all three Old World continents in the late eighteenth and early nineteenth century, suggesting that Africa was not a nutritional backwater in the years before more direct European involvement.^129^

As a reaction to the gendered pressures of colonial government, protracted breastfeeding and sexual abstinence were increasingly untenable throughout the twentieth century, with birth spacing durations declining almost universally across the continent.^130^ This was, in part, a result of colonial biopolitics. In the Belgian Congo, Africa’s most extreme example of engineered pronatalism, ‘birth bonuses’ worth 5 days’ pay were given to contracted labourers working on plantations and in mines across the colony.^131^ Even in less invasive spaces, such as the Gold Coast, state-sponsored baby shows formed a mechanism of ‘social regulation, if not social control’ as early as the 1920s. Mothers were encouraged to bring up children according to western ideals and rewarded with sugar, soap and children’s clothes when these conditions were met.^132^ The systemic influence of capitalist development compounded such policies. Again in the Gold Coast, male ownership of extra-subsistence produce severely undermined the value of childbearing, childrearing and food production, the biologically and socially ascribed outputs of female labour.^133^ Accompanying the pervasive devaluation of such labour was a similarly pervasive pattern of gendered conflict.^134^ While domestic pressures reflected the various ideals of colonial governments and the shifting and spatially-bounded demands of capital formation, domestic compromise was a fairly universal phenomenon across imperial Africa.

Endemic kwashiorkor could be utilised as a tool for colonial governance partly because the medicalisation of the disease stripped it of its social and economic context.^135^ Reducing kwashiorkor to a deficiency of protein allowed for its presentation as a failure on the part of African mothers, communities and cultures. In this respect, kwashiorkor helped justify European cultural hegemony and the paternalism of imperial government. As with other manifestations of African illness, kwashiorkor was explained in terms of deviance from metropolitan ideals.^136^ Even Cicely Williams used the existence of kwashiorkor to explain that ‘the idea that the “simple savage” has instinctive knowledge in caring for her children is without foundation’.^137^

It took two decades for ‘kwashiorkor’ to be accepted into the medical lexicon. When it was, the medicalised use of the word erased much of its original meaning at the same time as adding new import. A 1949 editorial in *The Lancet* explained that ‘“kwashiorkor” has the merit of neutrality: it offers no explanation and its use prejudices no issue’.^138^ The neutrality of the word was, however, sited entirely in the otherness of African language and its apparent incoherence, something which, ironically, entirely undermined any such neutrality. For the Ga, ‘kwashiorkor’ had a spiritual meaning which was not often said aloud. Williams had been stationed in the Gold Coast for 3 years before she heard the local name for a condition she had been seeing with some regularity.^139^ In 1960s Uganda, Mary Ainsworth expanded John Bowlby’s ‘attachment theory’ of child development to explain kwashiorkor in relation to *anorexia nervosa*, caused by the child’s perceived abandonment after the abrupt cessation of breastfeeding.^140^ Similar psycho-social definitions also underpinned Ga understandings of kwashiorkor. One doctor explained that kwashiorkor ‘applies to the psychological condition … in general conversation, if a child is crying, one might say to it, “what is your mother pregnant, are you getting kwashiorkor?”’^141^ In the 1930s, and apparently independent of Williams, the anthropologist Margaret Field described ‘*kwasiᴐkᴐ*’ as ‘a special kind of jealousy. Young children and babies can perceive far more than grown people and the first child soon begins to know that there is another one coming’.^142^ When the second baby is born, the first ‘resents the withdrawal of the mother’s attention … and may even die from sheer chagrin’. Children at risk were to be ‘treated with great patience, understanding, and humour’.^143^ In this context, kwashiorkor seems to have more properly suggested a broken taboo. Indeed, in the early 1960s, Akan respondents further north explained that ‘intercourse may affect the quality of the mother’s milk and it is said that many children die as a result of this … If such a thing happens, public opinion turns against the parents, especially the father; people say that he cannot control himself’.^144^ Whatever the exact definition, kwashiorkor was part of a complex system of social welfare based around religion, spirituality and those communalistic ideas of health which were often discarded with the ascendency of colonial medicine.

Under European government, indigenous knowledge was progressively devalued and replaced by biomedical frameworks that exalted scientific understandings of illness and promoted technical approaches to its relief. Michael Worboys has suggested that colonial interest in deficiency was notable because it ‘did not involve the creation of an exceptionalist, tropical nutritional science’.^145^ Yet such conclusions are not true of kwashiorkor. Following the popularisation of the Ga word, both the history and the terminology of the disease have tied protein deficiency specifically to the African continent. In their 1952 continent-wide survey for WHO, Brock and Autret explained that kwashiorkor is ‘a nutritional syndrome (or syndromes) found among indigenous Africans’, later explaining that ‘any clinical syndrome which includes these five characters and occurs in Africa can undoubtedly be called kwashiorkor’.^146^ Although they acknowledge that kwashiorkor may occur elsewhere, an African origin had become a core part of its pathology. The conceptualisation of kwashiorkor as an inherently African illness complemented an ahistorical understanding of the disease. Yarom and McFie’s 1963 conclusion, that ‘kwashiorkor has always been prevalent in the Kasai Province of South-East Congo’, denied the disease any history in the Congo, let alone a history relevant to its relief.^147^ This was part of a more general pattern which promoted Africa as a place of perpetual want. Speaking in Johannesburg in 1979, Jack Davies, the pathologist most closely concerned with the early biology of kwashiorkor, explained that the history of Africa had always been marred by ‘inadequacies of diet’. Davies went on to explain that ‘most of the foods which constitute the current dietary staples of African people have been developed elsewhere … it has puzzled some investigators as to just what were the dietary staples prior to the introduction of these foods’.^148^ The promotion of kwashiorkor as both the world’s gravest nutritional concern and as a uniquely African tropical disease sits comfortably within this narrative. Framing deficiency as Africa’s natural lot obstructs any critical appreciation of nutritional change. It also contributes to the fetishisation of an ahistorical form of African poverty entirely dissociated from the effects of colonial rule.

Considered ‘so common that many doctors would regard it as almost normal’, endemic kwashiorkor offered scientific and eugenicist justifications for colonial authority.^149^ Playing on long-held assumptions regarding the idleness of African populations, a continent-wide deficiency of protein went some way to explain African underdevelopment.^150^ In the Gold Coast, Purcell had explained that ‘the men of Akim are generally regarded as being weak-willed, lazy and cowardly … such inferiority may be attributed to their diet’.^151^ Brock and Autrets’ 1952 WHO report extended similar speculations across the continent, stating that ‘it would not be too far-fetched to attribute to that protein deficiency, at least in part, the backwardness of the African people’.^152^ Even in 1979, Jack Davies would ask his Johannesburg audience ‘how much did this nutritionally-induced apathy contribute to the docility of Negro slaves?’^153^

If protein deficiency naturalised African underdevelopment, whiggish understandings of economic and technological modernisation offered a reprieve. Working from the assumption that ‘cow’s milk [is] normally the most convenient source of protein for the child during the post-weaning period’, colonial veterinary services promoted the intensification of livestock cultivation.^154^ Although morbidity and mortality improved in cattle populations, limited tangible success left significant room for imported produce.^155^ Building on the model developed by Bovril, Oxo and other nineteenth-century nutraceuticals, similar solutions were sought to fill gaps in colonial diets and ply colonial markets. In a 1929 ‘Index to the Literature of Food Investigation’, 27 pages detail scientific advances in the manufacture, preservation and distribution of meat and animal products, 18 explore fruits and vegetables and 4 consider grains, crops and seeds. ‘At the charge of the Empire Marking Board’ these pamphlets were ‘distributed gratis to the Colonial Governments’.^156^ The promotion of breastmilk substitutes were part of this broader trend. In the Gold Coast, Purcell explained that ‘the condition of the infants indicate strongly that breastmilk is often of poor quality’. However, at the Oda Weighing Clinic, bottle-fed Baby Kofi ‘differed from all the other babies in that he was plump, robust and constantly cheerful.… The healthy gleam of his eyes was sufficient to distinguish him from the other babies, all of them breast-fed’.^157^ In 1937, only 2 years prior to her ‘milk and murder’ speech, even Cicely Williams would recommend that ‘milk should be given with every feed until a child is eighteen months old and every day till he is ten years old’.^158^ In fact, Williams was so optimistic about the potential for artificial feeding that she sought to acquire an official endorsement of Nestlé’s tinned milk.^159^

## Conclusion

Cicely Williams’ early faith in the potential of infant formula was cultured in both metropolitan and imperialist discourses regarding the relative value of food. In the 1930s, the science of nutrition pledged an objective valuation of diet, while technological developments appeared to offer a ready reprieve from deficiency. These ideas underpinned a biopolitics of nutrition that was based on earlier histories of class and cuisine, hunger, humanitarianism and *noblesse oblige*. Born from these conventions, kwashiorkor was taken to be a natural result of the deviant diets and food cultures encountered by Europeans in Africa. Made endemic both by the pressures of colonisation and the reification of nutritionist dietetics, kwashiorkor was cast as a pervasive and timeless burden of African incivility. While this construction was not apolitical, it reflects the latent politics of European history on the later development of colonial medicine.

Working from these assumptions, and working out of atypical rainforest food economies, early-twentieth-century nutrition research could only ever offer partial insight into the aetiology and epidemiology of deficiency during this period. In spite of this, conclusions drawn from these areas were extended into savannah areas and then across the continent. *Gold Coast Nutrition and Cookery*, like the infant formula which it endorsed, was one of many solutions to this construction of deficiency. Taking up this mantel, the UN’s Protein Advisory Group, created in 1955 to ‘fight to close the protein gap’ between Global North and South, continued to promote modernist, technical and technocratic solutions to an imagined protein crisis until long after the end of empire.^160^ It was only in 1975, in a word-for-word reversal of the conclusions forwarded in *Nutrition in the Colonial World*, that *Nature* carried an article explaining that ‘the problem is mainly one of quantity rather than quality of food’, and that ‘the protein gap is a myth … what really exists, even for vulnerable groups, is a food gap and an energy gap’.^161^

Kwashiorkor had emerged and endured also because its construction covered this up. Given that ‘no-one may starve in the British Empire’, kwashiorkor offered a generalisation of African deficiency which was politically benign. Unlike the more problematic politics of hunger and undernutrition, kwashiorkor actively bolstered the racialised hierarchies which lay at the foundations of empire. The apparent absence of kwashiorkor in European epidemiology—as well as its complex biological pathology—challenged the science of nutrition, justified European authority over African health and naturalised narratives of African exceptionalism. These singular narratives have been hard to shift and, as Chimamanda Ngozi Adichie has explained, ‘create stereotypes, and the problem with stereotypes is not that they are untrue, but that they are incomplete’.^162^ This does not mean to deny Africa’s burden of nutritional illness, or even the uniqueness of African ill-health, but seeks to instead stress that both malnutrition and African alterity have long conceptual histories relevant to contemporary problems; stories which were often spun in view of a metropolitan audience and, at times, for imperialist ends.

## Footnotes

1 See, for instance, Nancy Rose Hunt, *A Colonial Lexicon of Birth Ritual, Medicalization, and Mobility in the Congo* (Durham, NC: Duke University Press, 1999); Jean Allman, ‘Making Mothers: Missionaries, Medical Officers and Women’s Work in Colonial Asante, 1924-1945’, *History Workshop*, 1994, 38, 23–47.

2 Gold Coast Government, *Gold Coast Nutrition and Cookery* (Edinburgh: Published for the Gold Coast Government by T. Nelson and Sons, 1953), 270–71.

3 *Ibid*., 280.

4 For economic and social changes to domestic reproduction under colonial rule, see Claude Meillassoux, *Maidens, Meal and Money: Capitalism and the Domestic Community* (Cambridge: Cambridge University Press, 1981); Jean Comaroff and John L. Comaroff, ‘Home-Made Hegemony: Modernity, Domesticity, and Colonialism in South Africa’, in Karen Hansen, ed., *African Encounters with Domesticity* (New Brunswick, N.J.: Rutgers University Press, 1992), 37–74; for the nutritional effects, see Henrietta L. Moore and Megan Vaughan, *Cutting Down Trees: Gender, Nutrition, and Agricultural Change in the Northern Province of Zambia, 1890-1990* (London: James Currey, 1994); John Nott, ‘Malnutrition in a Modernising Economy: The Changing Aetiology and Epidemiology of Malnutrition in an African Kingdom, Buganda c.1940–73’, *Medical History*, 2016, 60, 229–49.

5 D. B. Jelliffe, ‘Pediatrics in Uganda’, *Clinical Pediatrics*, 1965, 4, 55–61.

6 D. B. Jelliffe and F. J. Bennett, ‘Cultural and Anthropological Factors in Infant and Maternal Nutrition’, *Federation Proceedings*, 1961, 20, S185–87.

7 H. F. Welbourn, ‘Bottle Feeding: A Problem of Modern Civilization’, *Journal of Tropical Pediatrics*, 1958, 3, 157–66; Nott, ‘Malnutrition’.

8 Cicely D. Williams, *“Milk and Murder”: Reprint of a Speech given to the Singapore Rotary Club, 1939* (Penang, Malaysia: International Organization of Consumers Unions, 1986), 5.

9 D. B. Jelliffe, ‘Commerciogenic Malnutrition?’, *Nutrition Reviews*, 1972, 30, 199–205.

10 On the scale of nutrition mortality in contemporary contexts, see Robert E. Black *et al.*, ‘Maternal and Child Undernutrition and Overweight in Low-Income and Middle-Income Countries’, *The Lancet*, 2013, 382, 427–51; for the current relationship between breastmilk substitutes and malnutrition, see Ellen G. Piwoz and Sandra L. Huffman, ‘The Impact of Marketing of Breast-Milk Substitutes on WHO-Recommended Breastfeeding Practices’, *Food and Nutrition Bulletin*, 2015, 36, 373–86; for the historical context of these trends, see Tehila Sasson, ‘Milking the Third World? Humanitarianism, Capitalism, and the Moral Economy of the Nestlé Boycott’, *The American Historical Review*, 2016, 121, 1196–224; John Nott, ‘“How Little Progress”? A Political Economy of Postcolonial Nutrition’, *Population and Development Review*, 2018, 60, 229–49.

11 Cicely D. Williams, ‘A Nutritional Disease of Childhood Associated with a Maize Diet’, *Archives of Disease in Childhood*, 1933, 8, 423–33, 432; Cicely D. Williams, ‘Kwashiorkor: A Nutritional Disease of Children Associated with a Maize Diet’, *The Lancet*, 1935, 226, 1151.

12 For the history of nutritional science, see, amongst others, Harmke Kamminga and Andrew Cunningham, eds, *The Science and Culture of Nutrition, 1840-1940* (Amsterdam: Brill Rodopi, 1995); E. C. Spary, *Feeding France: New Sciences of Food, 1760–1815* (Cambridge: Cambridge University Press, 2014).

13 For the history of the ‘protein deficit’ and the longer conceptual history of protein see, Kenneth J. Carpenter, *Protein and Energy* (Cambridge: Cambridge University Press, 1994).

14 For a general history of colonial medicine, see Megan Vaughan, *Curing Their Ills: Colonial Power and African Illness* (Stanford, CA: Stanford University Press, 1991); for the ‘discovery’ of malnutrition, see Michael Worboys, ‘The Discovery of Colonial Malnutrition Between the Wars’, in David Arnold, ed., *Imperial Medicine and Indigenous Societies* (Manchester: Manchester University Press, 1988), 208–25; David Arnold, ‘The “Discovery” of Malnutrition and Diet in Colonial India’, *The Indian Economic & Social History Review*, 1994, 31, 1–26; for the postcolonial history of nutrition in Africa, see Diana Wylie, *Starving on a Full Stomach: Hunger and the Triumph of Cultural Racism in Modern South Africa* (Charlottesville: University Press of Virginia, 2001); Cynthia Brantley, *Feeding Families: African Realities and British Ideas of Nutrition and Development in Early Colonial Africa* (Portsmouth, NH: Heinemann, 2002); Jennifer Tappan, *The Riddle of Malnutrition: The Long Arc of Biomedical and Public Health Interventions in Uganda* (Athens, OH: Ohio University Press, 2017).

15 Helen Tilley, *Africa as a Living Laboratory: Empire, Development, and the Problem of Scientific Knowledge, 1870-1950* (Chicago: University of Chicago Press, 2011).

16 The exceptionalism of African poverty has been touched on here, Michael Watts, ‘Entitlements or Empowerment? Famine and Starvation in Africa’, *Review of African Political Economy*, 1991, 51, 9–26; for the exceptionalism of African health, see Jean Comaroff, ‘The Diseased Heart: Medicine, Colonialism and the Black Body’, in Shirley Lindenbaum and Margaret M. Lock, eds, *Knowledge, Power, and Practice: The Anthropology of Medicine and Everyday Life* (Berkeley: University of California Press, 1993), 305–29.

17 Nolwazi Mkhwanazi, ‘Medical Anthropology in Africa: The Trouble with a Single Story’, *Medical Anthropology*, 2016, 35, 193–202.

18 F. I. D. Konotey-Ahulu, ‘Issues in Kwashiorkor’, *The Lancet*, 1994, 343, 548; on the unresolved history of famine, undernutrition and empire, see John Iliffe, *The African Poor: A History* (Cambridge: Cambridge University Press, 1987); Michael Watts, *Silent Violence: Food, Famine & Peasantry in Northern Nigeria* (Berkeley: University of California Press, 1983).

19 A history of nutrition research in Uganda has been a long time coming, and only properly addressed in Jennifer Tappan’s recent study. This paper re-treads some of this history, and at times with similar source material, although for very different ends. See Tappan, *Riddle*. The economic history of nutrition in southern Uganda can be found in Nott, ‘Malnutrition’; Jan Kuhanen, *Poverty, Health, and Reproduction in Early Colonial Uganda* (University of Joensuu: Faculty of Humanities, 2005).

20 Gyorgy Scrinis, *Nutritionism: The Science and Politics of Dietary Advice* (New York: Columbia University Press, 2013).

21 Michale Pollan, *In Defence of Food: The Myth of Nutrition and the Pleasures of Eating* (London: Allen Lane, 2008).

22 Worboys, ‘Discovery of Colonial Malnutrition’.

23 Tripp Rebrovick, ‘The Politics of Diet “Eco-Dietetics,” Neoliberalism, and the History of Dietetic Discourses’, *Political Research Quarterly*, 68, 2015, 678–89.

24 William Drennan, ‘To Ireland’, in John Aikin and Benson Earle Hill, eds, *The Monthly Magazine* (London: R. Phillips, 1796), 404, 404.

25 Steven Shapin, ‘How to Eat Like a Gentleman: Dietetics and Ethics in Early Modern England’, in C. Rosenberg, eds*, In Right Living: An Anglo-American Tradition of Self-Help Medicine and Hygiene* (Baltimore: Johns Hopkins University Press, 2003), 21–58; Rebrovick, ‘The Politics of Diet “Eco-Dietetics”', 681.

26 Carole Shammas, ‘The Eighteenth-Century English Diet and Economic Change’, *Explorations in Economic History*, 1984, 21, 254–69.

27 John Komlos, ‘Shrinking in a Growing Economy? The Mystery of Physical Stature During the Industrial Revolution’, *The Journal of Economic History*, 1998, 58, 779–802.

28 Kenneth J. Carpenter, ‘A Short History of Nutritional Science: Part 2 (1885–1912)’, *The Journal of Nutrition*, 2003, 113, 975–84.

29 George F. Shee, ‘The Deterioration in National Physique’, *Nineteenth Century*, 1903, 53, 797–805.

30 Richard Soloway, ‘Counting the Degenerates: The Statistics of Race Deterioration in Edwardian England’, *Journal of Contemporary History*, 1982, 17, 137–64, 142.

31 James Vernon, ‘The Ethics of Hunger and the Assembly of Society: The Techno-Politics of the School Meal in Modern Britain’, *The American Historical Review*, 2005, 110, 693–725; these were pan-European concerns which caught the attention of the continent’s foremost scientists. See Justus von Liebig, *Animal Chemistry, or Organic Chemistry in Its Applications to Physiology and Pathology* (London: Taylor and Walton, 1842).

32 Michel Foucault, *The History of Sexuality. Vol. 1: The Will to Knowledge* (London: Penguin, 1979), 135–45.

33 Robyn Smith, ‘The Emergence of Vitamins as Bio-Political Objects during World War I’, *Studies in History and Philosophy of Biological and Biomedical Sciences*, 2009, 40, 179–89.

34 Celia Petty, ‘Primary Research and Public Health: The Prioritisation of Nutrition Research in Inter-War Britain’, in J. Austoker and L. Bryder, eds, *Historical Perspectives on the Role of the MRC* (Oxford: Oxford University Press, 1989), 83–108.

35 Quoted in Deborah M. Valenze, *Milk: A Local and Global History* (New Haven: Yale University Press, 2011), 254.

36 Philip D. Curtin, *Death by Migration: Europe’s Encounter with the Tropical World in the Nineteenth Century* (Cambridge: Cambridge University Press, 1989), 125–29.

37 For detailed discussion of this important study, see Cynthia Brantley, ‘Kikuyu-Maasai Nutrition and Colonial Science: The Orr and Gilks Study in Late 1920s Kenya Revisited’, *The International Journal of African Historical Studies*, 1997, 30, 49–86; see also, Worboys, ‘Discovery of Colonial Malnutrition’.

38 J. B. Orr and J. L. Gilks, *Studies of Nutrition: The Physique and Health of Two African Tribes* (London: HMSO, 1931), 12.

39 Quoted in G. E. Metcalfe, *Great Britain and Ghana: Documents of Ghana History, 1807-1957* (London: Thomas Nelson & Sons for the University of Ghana, 1964), 613.

40 London School of Hygiene and Tropical Medicine archive, London, 0809/Nutrition/03, 'Volta River Project – Ghana.’

41 Public Records and Archives Administration Department (PRAAD), Accra, ADM/11/1/1294, F. M. Purcell, ‘Report of the Standing Committee to Study the Important Question of Human Nutrition, 1937–41’.

42 PRAAD/RG/3/5/600, A. Fulton, ‘Survey of Meat Supplies and Distribution in the Gold Coast.’

43 Brantley, ‘Kikuyu-Maasai’, 77.

44 See, Edward W. Said, *Orientalism* (London: Routledge & Kegan Paul, 1978).

45 Douglas M. Haynes, *Imperial Medicine: Patrick Manson and the Conquest of Tropical Disease* (Philadelphia: University of Pennsylvania Press, 2001), 140–51.

46 See, for example, W. E. McCulloch, *An Inquiry into the Dietaries of the Hausas and Town Fulani of Northern Nigeria, with Some Observations of the Effects on the National Health, with Recommendations Arising Therefrom* (Lagos: Government Printer, 1930); M. Fortes and S. L. Fortes, ‘Food in the Domestic Economy of the Tallensi’, *Africa*, 1936, 9, 237–76.

47 Quoted in Arnold, ‘The “Discovery”’, 12–13.

48 Orr and Gilks, *Studies of Nutrition*, 17.

49 Brantley, ‘Kikuyu-Maasai’, 80.

50 E. Burnet and W. R. Aykroyd, ‘Nutrition and Public Health’, *League of Nations: Quarterly Bulletin of the Health Organisation*, 1935, 4, 323–474, 452.

51 The National Archives, Kew (TNA) /CO/859/14/6, G.C. Eastwood, Joint Secretary to the Committee on Nutrition in the Colonial Empire, draft circular, 8 November 1939.

52 Malcolm Hailey, ‘Nutrition in the Colonies’, *The Times*, 26 July 1939.

53 TNA/CO/323/1571/5, ‘Nutrition in the British Colonial Empire: Summary of Replies’, November 1937.

54 Emily Klancher Merchant, ‘The Imperial Politics of (Mal)Nutrition in Colonial Gold Coast’ (Unpublished working paper, University of Michigan, 2005).

55 It should be recognised that the science of kwashiorkor is far from settled. See, for instance, M. H. Golden, ‘The Development of Concepts of Malnutrition’, *The Journal of Nutrition*, 2002, 132, 2117S–2122S; for a textbook discussion of PEM as understood today, see A. Stewart Truswell, ‘Protein-Energy Malnutrition’, in A. Stewart Truswell and Jim Mann, eds, *Essentials of Human Nutrition* (Oxford: Oxford University Press, 2012), 301–09; for current approaches to the management of malnutrition, see Steve Collins *et al.*, ‘Management of Severe Acute Malnutrition in Children’, *The Lancet*, 2006, 368, 1992–2000; for historical context, see Nott, ‘“How Little Progress”?’

56 See, for example, Bruno Latour, *Science in Action: How to Follow Scientists and Engineers Through Society* (Milton Keynes: Open University Press, 1987).

57 See, for example, Charles E. Rosenberg, ‘Disease in History: Frames and Framers’, *The Milbank Quarterly*, 1989, 670, 1–15.

58 For a discussion of retrospective diagnosis see, amongst others, Bruno Latour, ‘On the Partial Existence of Existing and Nonexisting Objects’, in Lorraine Daston, ed., *Biographies of Scientific Objects* (Chicago: University of Chicago Press, 2000), 247–69; Piers D. Mitchell, ‘Retrospective Diagnosis and the Use of Historical Texts for Investigating Disease in the Past’, *International Journal of Paleopathology*, 2011, 1, 81–88.

59 J. F. Brock and M. Autret, *Kwashiorkor in Africa* (Rome: FAO, Nutritional Studies, no. 8, 1952), 72.

60 H. F. Welbourn, ‘The Danger Period During Weaning’, *Journal of Tropical Pediatrics*, 1955, 1, 34–46; Valerie A. Fildes, *Breasts, Bottles and Babies: A History of Infant Feeding* (Edinburgh: Edinburgh University Press, 1985), 365–66.

61 Fildes, *ibid*., 390–93.

62 Edwin Clarke, ‘Whistler and Glisson on Rickets’, *Bulletin of the History of Medicine*, 1962, 36, 45–61, 48; John Graunt, *Natural and Political Observations Mentioned in a Following Index, and Made upon the Bills of Mortality* (London: Thomas Roycroft, 1662).

63 Stephen Nicholas and Deborah Oxley, ‘The Living Standards of Women During the Industrial Revolution, 1795-1820’, *The Economic History Review*, 1993, 46, 723–49.

64 See, for example, George Armstrong, *An Essay on the Diseases Most Fatal to Infants* (London: T. Cadell, 1771); Eustace Smith, *On the Wasting Diseases of Infants and Children* (London: J. & A. Churchill, 1868).

65 Rijpma, *Livingstone*.

66 A. Czerny and A. Keller, *Des Kindes Ernährung, Ernährungsstörungen Und Ernährungstherapie* (Leipzig: Franz Deuticke, 1906).

67 C.E. Bloch, ‘Diseases of Infants Due to Prolonged Feeding With Excess of Carbohydrates’, *British Medical Journal*, 1921, 1, 293–95; I.A. Abt, ‘Injuries Produced by Starch’, *Journal of the American Medical Association*, 1913, 61, 1275–77.

68 Jack Goody, *Cooking, Cuisine and Class: A Study in Comparative Sociology* (Cambridge: Cambridge University Press, 1982), 136.

69 Vincent J. Knapp, ‘The Democratization of Meat and Protein in Late Eighteenth- and Nineteenth-Century Europe’, *Historian*, 1997, 59, 541–51.

70 Jonathan Pereira, quoted in *Ibid.*, 546.

71 Mark R. Finlay, ‘Early Marketing of the Theory of Nutrition: The Science and Culture of Liebig’s Extract of Meat’, in Harmke Kamminga and Andrew Cunningham, eds, *The Science and Culture of Nutrition, 1840-1940* (Amsterdam: Brill Rodopi, 1995), 48–74; for the earlier French history see, Spary, 203–34; for analogous trends today see, for instance, Alice Street, ‘Food as Pharma: Marketing Nutraceuticals to India’s Rural Poor’, *Critical Public Health*, 2015, 25, 361–72.

72 Golden, S2117.

73 J.A. Nixon, ‘Famine Dropsy as a Food-Deficiency Disease’, *Bristol Medico-Chirurgical Journal*, 1920, 37, 137–48; A.D. Bigland, ‘Oedema as a Symptom in So-Called Food Deficiency Diseases’, *The Lancet*, 1920, 195, 243–47.

74 R. A. Frisch, Lafayette B. Mendel, and John P. Peters, ‘The Production of Edema and Serum Protein Deficiency in White Rats by Low Protein Diets’, *Journal of Biological Chemistry*, 1929, 84, 167–77.

75 A. Keys and others, *The Biology of Human Starvation* (Minneapolis: University of Minnesota Press, 1950), 921–65; R.A. McCance, ‘The History, Significance and Aetiology of Hunger Oedema’, in Department of Experimental Medicine, University of Cambridge, eds, *Studies of Undernutrition, Wuppertal 1946-9* (London: HMSO, 1951), 21–82.

76 Joseph Dupuis, *Journal of a Residence in Ashantee* (London: H. Colburn, 1824), v–vi.

77 T.M. Winterbottom, *An account of the native Africans in the neighbourhood of Sierra Leone* (London: C. Whittingham, 1803), 66.

78 Fortes and Fortes, 267.

79 F. M. Purcell, *Diet and Ill-Health in the Forest Country of the Gold Coast* (London: Lewis, 1939), 14.

80 M. Autret and M. Behar, *Síndrome Policarencial Infantil (Kwashiorkor) and Its Prevention in Central America* (Rome: FAO Nutritional Studies, no. 13, 1954).

81 L. Normet, ‘La Bouffissure d’Annam’, *Bulletin de La Société de Pathologie Exotique*, 1926, 3, 207–13.

82 R.A.W. Procter, ‘Medical Work in a Native Reserve’, *Kenya Medical Journal*, 1926, 3, 284–89.

83 J. Falkenstein, quoted in Sjoerd Rijpma, ‘Malnutrition in the History of Tropical Africa’, *Civilisations*, 1996, 43, 45–63.

84 Sjoerd Rijpma, *David Livingstone and the Myth of African Poverty and Disease: A Close Examination of His Writing on the Pre-Colonial Era* (Leiden: Brill, 2015), 436–37.

85 For the history of kwashiorkor’s ‘discovery’ see, H. C. Trowell, J. N. P. Davies, and R. F. A. Dean, *Kwashiorkor* (London: Edward Arnold, 1954); for a more critical history see, Tappan, *Riddle*, 11–36.

86 See, for instance, H. C. Trowell, ‘Infantile Pellagra’, *Transactions of The Royal Society of Tropical Medicine and Hygiene*, 1940, 33, 389–404.

87 Brock and Autret, *Kwashiorkor in Africa*, 21.

88 Trowell, ‘Infantile Pellagra’.

89 H. C. Trowell, J. N. P. Davies, and R. F. A. Dean, ‘Kwashiorkor—II. Clinical Picture, Pathology, and Diagnosis’, *British Medical Journal*, 1952, 2, 798–801.

90 Rhodes House, Oxford (RH)/MSS.Afr.S.1872, Box XX, J.N.P. Davies, *Personal Reminiscences*.

91 RH/MSS.Afr.S.1872, Box XXXV, H.C. Trowell, *Interview*, 1982.

92 *Ibid*.

93 Tappan, *Riddle*, 15.

94 ‘Nutrition in the British Colonial Empire’.

95 H. F. Welbourn, ‘Backgrounds and Follow-up of Children with Kwashiorkor’, *Journal of Tropical Pediatrics*, 1959, 5, 91.

96 Nutrition Sub-Committee, *Review of Nutrition in Uganda: A Summary of Previous Work and an Appreciation of the Present Position* (Entebbe: Government Printer, 1945), 3–5.

97 Wylie, *Starving on a Full Stomach*, 223.

98 Iliffe, *The African Poor*, 6; these ideas are not universally accepted. For instance, Watts takes the view that famine became more common if less severe under colonial government. Watts, *Silent Violence*.

99 Rijpma, *Livingstone*, 12.

100 This is even the case in very fine histories, see, for example, Wylie, *Starving on a Full Stomach*; Brantley, *Feeding Families;* James Vernon*, Hunger: A Modern History* (Cambridge, MA: Harvard University Press, 2007).

101 Worboys, ‘Discovery of Colonial Malnutrition’, 220.

102 PRAAD/ADM/11/1/1294, F. M. Purcell, ‘Report.’

103 The fascinating history of the Purcell survey is also detailed here, Jerôme Destombes, *Nutrition and Economic Destitution in Northern Ghana, 1930-1957. A Historical Perspective on Nutritional Economics*, Economic History Working Papers (London: Department of Economic History, LSE, 1999), pp. 1–63

104 TNA/CO/859/68/1, A.I. Richards, ‘Notes on Dr. Purcell’s reports on Nutrition in the Gold Coast, 1940’, 17 September 1941.

105 PRAAD/ADM/11/1/1294, F. M. Purcell, ‘Report.’

106 F. M. Purcell, ‘The Gold Coast Government, the Colonial Office, and Nutrition: Facts of an Astonishing Colonial Episode, Letters to the Editor’, *West Africa*, 4 December 1943, p. 1095.

107 TNA/CO/859/115/3, Note by S. Culwick, 5 December, 1943.

108 TNA/CO/859/68/1, Note by S. Culwick regarding the *West Africa* article, 21 July 1943.

109 ‘Colonies and Calories’, *West African Review*, March 1946.

110 ‘Nutrition in West Africa’, *West African Review*, June 1946.

111 TNA/CO/859/115/3, Letter from Sir Alan Burns, Governor of the Gold Coast, to Oliver Stanley, Secretary of State for the Colonies, 16 February, 1944.

112 Vernon, *Hunger*, 18–80.

113 TNA/CO/323/1570/7, Response from the Gold Coast government to a circular requesting ‘A summary of information regarding nutrition in the Colonial Empire’, 29 June 1938.

114 Worboys, ‘Discovery of Colonial Malnutrition’, 218.

115 A. I. Richards, *Land, Labour and Diet in Northern Rhodesia: An Economic Study of the Bemba Tribe* (London: Oxford University Press, 1939), 3; see also Monica Hunter, *Reaction to Conquest: Effects of Contact with Europeans on the Pondo of South Africa* (Oxford: Oxford University Press, 1936).

116 Worboys, ‘Discovery of Colonial Malnutrition’, 221–23.

117 RH/MSS.Afr.S.1872, Box XXXV, Trowell, *Interview*.

118 Jennifer Tappan, ‘“A Healthy Child Comes from a Healthy Mother”: Mwanamugimu and Nutritional Science in Uganda, 1935-1973’ (unpublished PhD thesis, Columbia University, 2010), 99–106.

119 Tilley, *Africa as a Living Laboratory*.

120 PRAAD/ADM/11/1/1294, F.M. Purcell, ‘Report.’

121 Steven Feierman and John M. Janzen, eds, *The Social Basis of Health and Healing in Africa* (Berkeley, CA: University of California Press, 1992).

122 Worboys, ‘Discovery of Colonial Malnutrition’, 222.

123 J. Stanton, ‘Listening to the Ga: Cicely Williams’ Discovery of Kwashiorkor on the Gold Coast’, *Clio Medica*, 2001, 61, 149–71; Sally Craddock, *Retired Except on Demand: The Life of Doctor Cicely Williams* (Oxford: Green College, 1983).

124 Williams, ‘Kwashiorkor’, 1151.

125 Cicely D. Williams, ‘The Mortality and Morbidity of the Children of the Gold Coast’ (unpublished MD thesis, University of Oxford, 1936), 35.

126 Williams, ‘Kwashiorkor’; Hugh S. Stannus, ‘Kwashiorkor’, *The Lancet*, 1935, 226, 1207–08, 1207.

127 Trowell, Davies, and Dean, *Kwashiorkor*, 283.

128 Quoted in, Rijpma, *Livingstone*, 447.

129 Joerg Baten and Matthias Blum, ‘Growing Tall But Unequal: New Findings and New Background Evidence on Anthropometric Welfare in 156 Countries, 1810–1989’, *Economic History of Developing Regions*, 2012, 27, S66–85; Gareth Austin, Joerg Baten, and Bas Van Leeuwen, ‘The Biological Standard of Living in Early Nineteenth-Century West Africa: New Anthropometric Evidence for Northern Ghana and Burkina Faso’, *The Economic History Review*, 2012, 65, 1280–302.

130 R. Schoenmaeckers *et al.*, ‘The Child-Spacing Tradition and the Postpartum Taboo in Tropical Africa: Anthropological Evidence’, in H. J. Page and R. Schoenmaeckers, eds, *Child-Spacing in Tropical Africa: Traditions and Change* (New York: Academic Press, 1981), 25–71.

131 Hunt, *A Colonial Lexicon of Birth Ritual*, 237–38.

132 Allman, ‘Making Mothers’, 23.

133 Jean Allman and Victoria Tashjian, *‘I Will Not Eat Stone’: A Women’s History of Colonial Asante* (Oxford: James Currey, 2000).

134 See, for example, Jean Allman, ‘Rounding up Spinsters: Gender Chaos and Unmarried Women in Colonial Asante’, *Journal of African History*, 1996, 37, 195–214; Margrethe Silberschmidt, *‘Women Forget That Men Are the Masters’: Gender Antagonism and Socio-Economic Change in Kisii District, Kenya* (Uppsala: Nordiska Afrikainstitutet, 1999).

135 For a more detailed discussion of the medicalisation of malnutrition see, Tappan, *Riddle*, 37–67.

136 Vaughan, *Curing Their Ills*, 1–29.

137 Cicely D. Williams, ‘Child Health in the Gold Coast’, *The Lancet*, 1938, 231, 97–102, 99.

138 ‘Kwashiorkor’, *The Lancet*, 1949, 253, 188–89, 188.

139 Craddock, *Retired Except on Demand*, 62.

140 Mary D. Salter Ainsworth, *Infancy in Uganda: Infant Care and the Growth of Love* (Baltimore: Johns Hopkins Press, 1967).

141 Wellcome Library (WL), London, PP/CDW/L.1, G. Saunders to M. Autret, 9 October 1953.

142 M. J. Field, *Religion and Medicine of the Gã People* (Oxford: Oxford University Press, 1937), 165. Although publishing after Williams, it seems Field is unaware of Williams’ work. She also has some experience in mental health, working at Maudsley Hospital ‘on the Biochemical aspect of Mental Pathology’ in 1927 (Field, v).

143 *Ibid*., 177.

144 Barrington Kaye, *Bringing up Children in Ghana: An Impressionistic Survey* (London: George Allen & Unwin, 1962), 67.

145 Worboys, ‘Discovery of Colonial Malnutrition’, 222.

146 Brock and Autret, *Kwashiorkor in Africa*, 11, 30.

147 R. Yarom and J. McFie, ‘Kwashiorkor in the Congo: A Clinical Survey of a Hundred Successive Cases in the Kasai Province’, *The Journal of Tropical Pediatrics and Environmental Child Health*, 1963, 9, 56–63, 56.

148 J. N. P. Davies, *Pestilence and Disease in the History of Africa* (Johannesburg: Witwatersrand University Press, 1979), 4, 7–8.

149 Brock and Autret, *Kwashiorkor in Africa*, 34.

150 Klas Rönnbäck, ‘The Idle and the Industrious – European Ideas about the African Work Ethic in Precolonial West Africa’, *History in Africa*, 2014, 41, 117–45.

151 Quoted in Trowell, Davies, and Dean, *Kwashiorkor*, 243.

152 Brock and Autret, *Kwashiorkor in Africa*, 32–33.

153 Davies, *Pestilence and Disease*, 8.

154 Brock and Autret, *Kwashiorkor in Africa*, 58.

155 K. David Patterson, ‘The Veterinary Department and the Animal Industry in the Gold Coast, 1909-1955’, *The International Journal of African Historical Studies*, 1980, 13, 457–91; the relationship between nutritional science and the development of both human and animal capital deserves more work, especially given the recent ‘animal turn’ in historiography. An interesting exception is, Jonathan Saha, ‘Milk to Mandalay: Dairy Consumption, Animal History and the Political Geography of Colonial Burma’, *Journal of Historical Geography*, 2016, 54, 1–12.

156 PRAAD/CSO/11/1/337, ‘Index to the literature of food investigation’, 1929.

157 Purcell, *Diet and Ill-Health*, 15.

158 WL/PP/CDW/B.1/3, Gold Coast Handbook of Nursing, Children's Section, Accra, 1937.

159 PRAAD/CSO/11/6/4, Cicely D. Williams to Deputy Director of Health Services, 18 January 1932.

160 Quoted in Carpenter, *Protein and Energy*, 162; see also Tom Scott-Smith, ‘Beyond the “Raw” and the “Cooked”: A History of Fortified Blended Foods’, *Disasters*, 2015, 39, 244–60; Nott, ‘“How Little Progress”?’

161 J. C. Waterlow and P. R. Payne, ‘The Protein Gap’, *Nature*, 1975, 258, 113–17, 113, 117.

162 Quoted in Mkhwanazi, ‘Medical Anthropology in Africa', 194.

## Notes

John Nott is a Postdoctoral Fellow in the STS Research Group at Maastricht University. He is currently writing a monograph on the long history of food and health in Ghana since the late nineteenth century.

